# Identification of gene expression models for laryngeal squamous cell carcinoma using co-expression network analysis

**DOI:** 10.1097/MD.0000000000009738

**Published:** 2018-02-16

**Authors:** Chun-wei Yang, Shu-fang Wang, Xiang-li Yang, Lin Wang, Lin Niu, Ji-Xiang Liu

**Affiliations:** a Department of Otorhinolaryngology Head and Neck Surgery, Tianjin Union Medical Center; b Intensive Care Unit, General Hospital Airport Hospital, Tianjin Medical University, Tianjin, China.

**Keywords:** biomarker, disease progression, Hub gene, LSCC, WGCNA

## Abstract

One of the most common head and neck cancers is laryngeal squamous cell carcinoma (LSCC). LSCC exhibits high mortality rates and has a poor prognosis. The molecular mechanisms leading to the development and progression of LSCC are not entirely clear despite genetic and therapeutic advances and increased survival rates. In this study, a total of 116 differentially expressed genes (DEGs), including 11 upregulated genes and 105 downregulated genes, were screened from LSCC samples and compared with adjacent noncancerous. Statistically significant differences (log 2-fold difference > 0.5 and adjusted *P-*value < .05) were found in this study in the expression between tumor and nontumor larynx tissue samples. Nine cancer hub genes were found to have a high predictive power to distinguish between tumor and nontumor larynx tissue samples. Interestingly, they also appear to contribute to the progression of LSCC and malignancy via the Jak-STAT signaling pathway and focal adhesion. The model could separate patients into high-risk and low-risk groups successfully when only using the expression level of mRNA signatures. A total of 4 modules (blue, gray, turquoise, and yellow) were screened for the DEGs in the weighted co-expression network. The blue model includes cancer-specific pathways such as pancreatic cancer, bladder cancer, nonsmall cell lung cancer, colorectal cancer, glioma, Hippo signaling pathway, melanoma, chronic myeloid leukemia, prostate cancer, and proteoglycans in cancer. Endocrine resistance (CCND1, RAF1, RB1, and SMAD2) and Hippo signaling pathway (CCND1, LATS1, SMAD2, and TP53BP2) could be of importance in LSCC, because they had high connectivity degrees in the blue module. Results from this study provide a powerful biomarker discovery platform to increase understanding of the progression of LSCC and to reveal potential therapeutic targets in the treatment of LSCC. Improved monitoring of LSCC and resulting improvement of treatment of LSCC might result from this information.

## 1 Introduction

With an incidence of 2.4% every year around the world, laryngeal squamous cell carcinoma (LSCC) is the most prevalent malignancy,^[1,2]^ corresponding to about 25% of head and neck squamous cell carcinoma (HNSCC) cases. Two main risk factors affecting the development of LSCC are tobacco use and alcohol consumption. Poor oral hygiene, nutritional deficiencies, and certain viruses are also notable risk factors.^[3–5]^ Due to the vital functions of the larynx in respiration and phonation, LSCC can seriously affect the daily life of patients.^[6]^

Therapy is mainly determined by age, performance, stage of disease, and tumor location. These factors are not sufficient to determine prognostic characterization of LSCC as patients with similar clinical features might be at a different stage of disease and thus have a different response to the therapy.^[7]^ Treatment of primary LSCC has little effect on patients with advanced stage disease, despite the use of advanced surgical intervention, chemotherapy, and radiotherapy.^[3]^ The long-term survival rate of patients remains at 50%, with high rates of associated mortality in HNSCC.^[4]^ There is still a pressing need to explore the molecular mechanisms causing LSCC development and progression in order to develop novel therapeutic schedules.

It has been demonstrated that cyclin-dependent kinase inhibitor 2A point mutation is linked to disease relapse and mortality. In fact, it could be a key biomolecular indicator in LSCC.^[8,9]^ According to Langfelder, the downregulated human leukocyte antigen (HLA) class I reduces the survival time of LSCC patients, thus allowing it to possibly serve as an independent prognostic marker.^[10]^ The overexpression of cyclin D1 and/or the overexpression of cyclin-dependent kinase 4 reveals the biological behavior of LSCC and is thus useful in the prognosis of the disease.^[11,12]^ Expression of S100 calcium binding protein A2, in Lu's study, is associated with cell commitment to squamous differentiation, cytokeratin expression, and overall survival in LSCC.^[13]^ The growth and invasion of LSCC, which can be used in the gene therapy of LSCC, can be inhibited by recombinant lentivirus mediated siRNA silencing of matrix metallopeptidase 2.^[14,15]^ Cao et al^[16,17]^ found that in correlation with clinical stage in humans, LSCC overexpressed stomatin-like protein 2 can promote cell growth, tumorigenicity, and adhesion. The mechanisms of LSCC remain unclear, in spite of studies performed to investigate LSCC.

In order to improve the prognosis and therapy of LSCC, identification of cancer-associated molecules and signaling networks is investigated in this study. The microarray of GSE10288 was analyzed. Screened from LSCC samples compared with adjacent noncancerous samples were a total of 116 DEGs, including 11upregulated genes and 105 downregulated genes. LSCC-specific gene co-expression networks were constructed by differential expression analysis and weighted gene co-expression network analysis (WGCNA). The study identified important pathways and putative cancer hub genes contributing to tumorigenesis of LSCC. An elastic-net regularized classification model was built using the cancer hub gene signatures to predict the early diagnosis of LSCC.

## 2 Materials and methods

### 2.1 Microarray data

The expression profile of GSE10288 was downloaded from the Gene Expression Omnibus (http://www.ncbi.nlm.nih.gov/geo/) and based on the platforms of GPL6426 Head and Neck carcinoma cDNA microarray. A collection of 26 LSCC tissue samples and 20 adjacent noncancerous tissue (ANT) samples was included in GPL6426.

### 2.2 DEG screening

Normalized microarray data were obtained when GSE10288 was downloaded from the Gene Expression Omnibus. The average value of the probes with low expression ≤0.1 was excluded. An average value was adopted if there existed duplicated genes. Subsequently, the linear models for microarray data (LIMMA) package were used to screen the DEGs between the LSCC samples and adjacent noncancerous samples. (http://www.bioconductor.org). The adjusted *P*-value of < .05 and **|**log fold change (FC)**| >** 0.5 ^[18]^ were used as the cut-off criteria.

### 2.3 Weighted co-expression network construction

Constructed with WGCNA package in *R* Pearson's correlation coefficients between the DEGs were calculated by their expression matrices constructed for weighted co-expression networks for the DEGs. The correlation coefficient of ≥0.8 was defined as the weighting coefficient. Using the hybrid dynamic shear tree method, a hierarchical clustering tree, branches of which represented the gene modules, was constructed for the DEGs.^[19]^ Involvement with at least 10 genes was required of each gene module. At that point, the calculation of the feature vector for each module and cluster analysis for the modules was performed. The closed modules (difference of feature vectors < 0.15) were merged into new modules. Additionally, correlation analysis between LSCC and modules was performed. The gene significance (GS) and the module significance (MS) were then calculated.^[18]^

### 2.4 Pathway enrichment analysis

The Kyoto Encyclopedia of Genes and Genomes (KEGG) is the background knowledge base related to systems information, genomic information, and chemical information.^[20]^ The KEGG pathway enrichment analysis was conducted separately for the DEGs in the modules by the cluster profiler package (http://bioconductor.org/packages/release/bioc/html/clusterProfiler.html). The cut-off criterion used was an adjusted *P-*value of < .05.^[18]^

### 2.5 Identification of cancer hub genes

Scaled connectivity (K) and GS were calculated using WGCNA. In the process of identification of the cancer hub genes, K and GS were implemented to take the LSCC-specific gene co-expression networks into account. WGCNA was used to identify the cancer hub genes that functionally contribute to the tumorigenesis of LSCC.

### 2.6 Diagnosis model construction

A logistic regression model was fit to the data to model the probability of malignant versus benign LSCC for each hub gene. Odds ratios were obtained with 95% confidence intervals using this model. The receiver operating characteristic (ROC) was constructed to estimate the area under the curve (AUC) as a measure of the overall diagnostic ability.

### 2.7 Statistical analysis

Statistical analysis was performed using R software (version 3.2.3) and whereas ROC analysis was utilized to evaluate the diagnostic value of hub genes in LSCC. A classification model of combined hub gene was built to evaluate discriminatory capacity of LSCC and non-LSCC, using linear logistic regression of hub genes.

## 3 Results

### 3.1 DEG analysis

**Figure 1:**
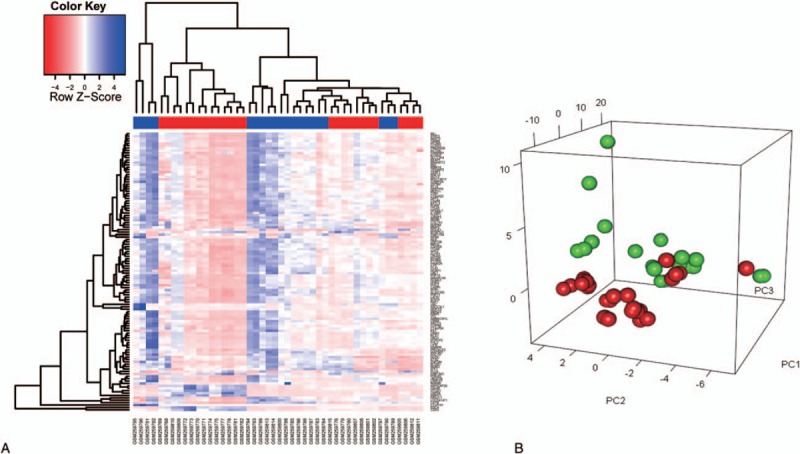
(A), Identification consensus DEGs screening of training and test set in patients with laryngeal squamous cell carcinoma. The 116 DEGs LSCC (red label) vs ANT (green label) of GSE10288 set. Each column is a sample and each row is the expression level of an mRNA. The color scale represents the raw *Z*-score ranging from blue (low expression) to red (high expression). Dendrograms beside each heatmap correspond to the hierarchical clustering by expression of the 116 DEGs. (B) PCA plot showing complete, unsupervised separation of the 46 array samples into 26 LSCC (red) and 20 ANT (green) samples. ANT = adjacent noncancerous tissue, DEGs = differentially expressed genes, LSCC = aryngeal squamous cell carcinoma, PCA = principal component analysis.

Around 116 DEGs were separated by LIMMA from the LSCC samples, including 11 upregulated and 105 downregulated genes, when compared with the adjacent noncancerous samples. The results showed that most of dysregulated genes in LSCC are reduced. The ability to separate LSCC samples from ANTs in the 2-way hierarchical cluster (Fig. 1A and B) involved the mRNA signature.

### 3.2 Weighted co-expression network construction

**Figure 2:**
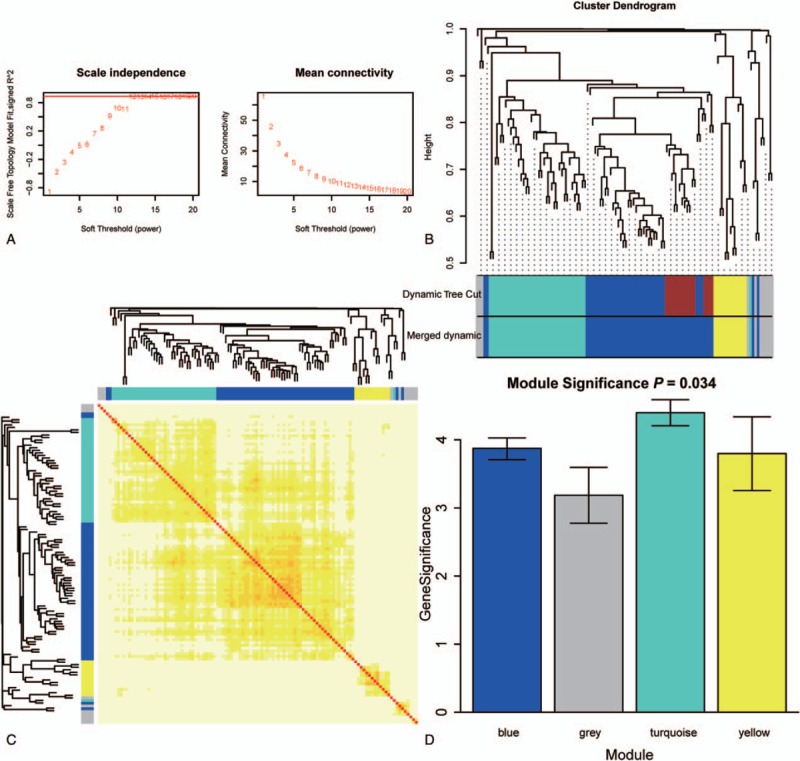
Network construction of weighted co-expressed genes. (A) Selection of the weighting coefficient. (B) Hierarchical clustering tree of the DEGs. In the hierarchical dendrogram, lower branches correspond to higher co-expression (height = Euclidean distance). (C) Heatmap view of topological overlap of co-expressed genes in different modules. The heatmap was generated from topological overlap values between genes. The genes were grouped in modules labeled by a color code, which are given under gene dendrogram on both sides. The topological overlap is high among genes of same module. (D) Distribution of the average gene significance and errors in the modules associated with LSCC. LSCC = laryngeal squamous cell carcinoma.

The weighted co-expression network and the weighting coefficient were constructed and set as 12 (Fig. 2A). Modules were identified from the weighted co-expression network. A total of 4 modules (blue, gray, turquoise, and yellow modules) were screened for the DEGs after these closed modules were merged (Fig. 2B). After the highly similar modules were merged, a total of 4 co-expressed modules were identified, ranging from 10 to 54 genes, while the gray module was reserved for genes that were not co-expressed (Fig. 2C). To verify the correlation between ME and the LSCC, a measure of module significance, the average gene significance of all of the genes was calculated. Module turquoise showed the highest mean gene significance indicating that genes in module turquoise may play an important role in affecting LSC according to the distribution of the gene significance in all modules (Fig. 2D).

### 3.3 Hub genes of turquoise module and early diagnosis model

In order to determine hub genes or genes that were the most connected to other genes and individually significant genes, intramodular analysis was performed. A hub gene was defined as a gene with gene significance (GS) ≥ −log_10_ (0.05) and module membership (MM) ≥ 0.95. It was revealed that there were 9 LSCC hub genes in this turquoise cluster (ALS2CR3, ALPK1, STAT3, PHF11, SART3, SIDT2, MLL, RYR1, and MED11) (Table 1).

**Table 1:**
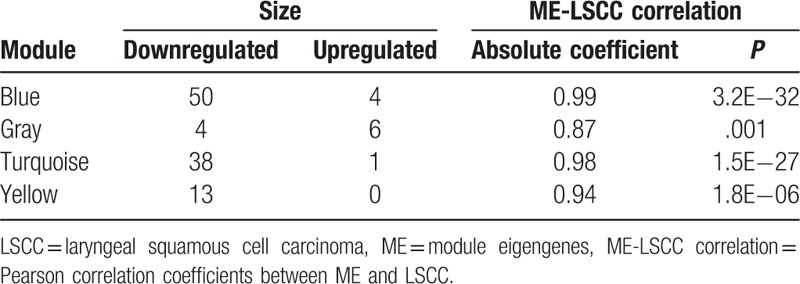
Statistics for the 4 modules (blue, gray, turquoise, and yellow modules).

**Figure 3:**
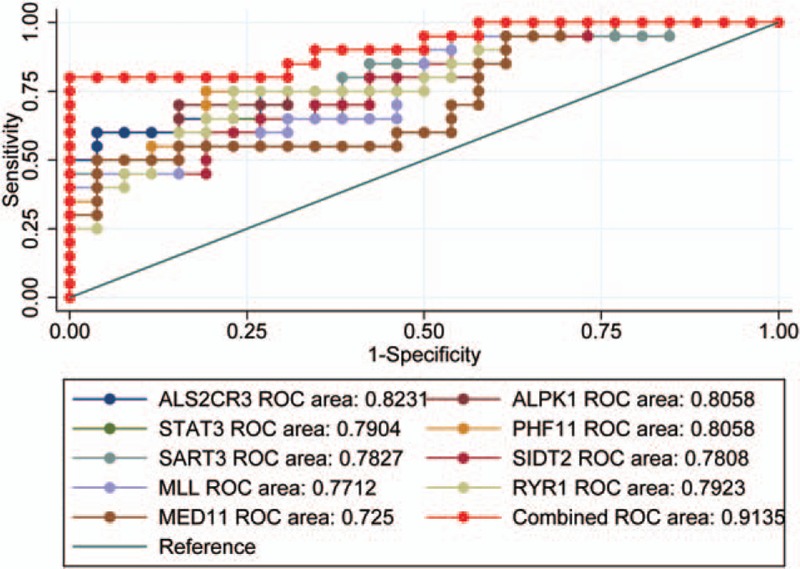
ROC curve to assess the accuracy of the model composed of the cancer hub genes signature. True positive rate represents the model sensitivity, whereas false positive rate is one minus the specificity or true negative rate and represents chance. ROC = receiver operating characteristic.

Receiver operating characteristic (ROC) curves were built to estimate the performance of 9 hub genes combined model between LSCC and non-LSCC to further investigate whether the 9 hub genes correlate with the early diagnosis of LSCC patients. AUC values for 9 hub genes were greater than 0.7. By employing a linear regression model built on a panel of the combined 9 hub genes (AUC = 0.744): risk score = 0.369 × SCG3 + 0.296 × SYP + 0.128 × RUNDC3A −0.073 × CDK5R2 + 0.115 × AP3B2 −3.55, we could attain a best performance on accuracy (Fig. 3).

### 3.4 Pathway enrichment analysis

Pathway enrichment analyses were conducted separately for the DEGs in each module by using the cluster profiler package. Only the DEGs in the blue, turquoise, and yellow modules were involved in pathways (Table 2**)**. For the DEGs in the yellow module, the enriched pathway was the tumor necrosis factor signaling pathway, transcriptional disregulation in cancer, HTLV-I infection, platinum drug resistance, IL-17 signaling pathway, and endocrine resistance. For the DEGs in the blue module, including pancreatic cancer (*P* = 4.13×10^−5^), bladder cancer (*P* = 2.43×10^−4^), and nonsmall cell lung cancer (*P* = 6.14×10^−4^), there were 12 enriched pathways. Of particular interest, and exhibiting high connectivity degrees in the blue module were cyclin D1 (CCND1; degree, 11), Raf proto-oncogene serine/threonine-protein kinase (RAF1; degree, 10), RB transcriptional corepressor 1 (RB1; degree, 8), and SMAD family member 2 (SMAD2; degree, 4). The enriched pathway was the Jak-STAT signaling pathway, focal adhesion, inflammatory bowel disease, regulation of actin cytoskeleton, adherens junction, and prolactin signaling pathway for the DEGs in the turquoise module.

**Table 2:**
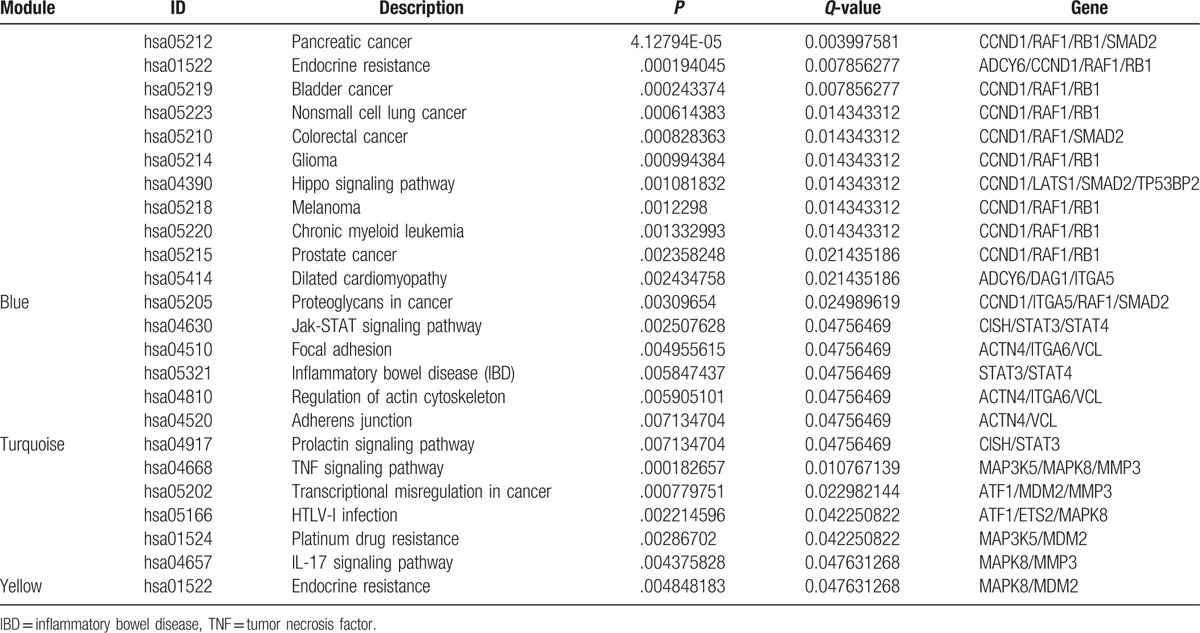
Pathways enriched for differentially expressed genes in the blue, turquoise, and yellow modules.

## 4 Discussion

In this study, a total of 116 DEGs, including 11 upregulated genes and 105 downregulated genes were screened from LSCC samples and compared with adjacent noncancerous samples. All 116 genes showed statistically significant differences (log 2-fold difference >0.5 and adjusted *P-*value < .05) in the expression between tumor and nontumor larynx tissue samples. Nine cancer hub gene signatures exhibited high predictive power to distinguish between tumor and nontumor larynx tissue samples. Interestingly, seeming to contribute to LSCC progression and malignancy via Jak-STAT signaling pathway and focal adhesion in turquoise module were these hub gene networks. This model could separate patients into high- and low-risk groups successfully only using the expression level of mRNA signatures.

In the weighted co-expression network, a total of 4 modules (blue, gray, turquoise, and yellow modules) were screened for DEGs. The DEGs in the blue module were involved in cancer-specific pathways, including pancreatic cancer, bladder cancer, nonsmall cell lung cancer, colorectal cancer, glioma, Hippo signaling pathway, melanoma, chronic myeloid leukemia, prostate cancer, and proteoglycans in cancer. Perhaps of great importance in LSCC, as it had high connectivity degrees in the blue module, is the endocrine resistance (CCND1, RAF1, RB1, and SMAD2), Hippo signaling pathway (CCND1, LATS1, SMAD2, and TP53BP2).

One of the most studied cyclins is CCDN1. Controlling the cell cycle through forming complexes with cyclin-dependent kinases and hence activating them, is cyclin, a highly conserved protein. These complexes function as regulators with an important periodicity through the different phases of the cell cycle. Protein expression levels of CCND1 are related to the site of the tumor, depth of tumor invasion, and stage of the disease. Furthermore, associated with the existence of LSCC located in the supraglottic larynx, highly invasive (T3 and T4) malignant neoplasms of the larynx and tumors being at an advanced clinical stage (III and IV) are CCND1 immunopositivities. In addition, the overexpression of CCND1 was linked to positive nodal.^[21–23]^ Besides CCND1 protein over-expression, high CCND1 mRNA expression was also associated with local invasion and stage IV LSCC. Measured in several LSCC specimens, it has been suggested that CCND1 gene amplification could account for high CCND1 mRNA levels.^[24]^

LATS1 is also known as WARTS, is an enzyme that in humans is encoded by the LATS1 gene. It has been associated with the Hippo signaling pathway.^[25]^ Involved in the regulation of various cellular processes, LATS1 encodes a serine/threonine kinase. A tumor-suppressing pathway called the Salvador–Warts–Hippo (SWH) pathway, was revealed by genetic studies in *Drosophila* which have identified LATS as a central mediator.^[26,27]^ Also a critical factor in the regulation of organ size in *Drosophila melanogaster* and mamma is the SWH pathway.^[28,29]^ Further, implicated in the genesis of multiple human cancers is the deregulation of SWH pathway activity.^[30–32]^ LATS1 plays a suppressor role in some tumors. Moreover, decreased LATS1 expression was observed in breast, cervical, and head and neck squamous cell cancers.^[33]^

The first tumor suppressor gene to be molecularly defined was the retinoblastoma susceptibility gene (RB1). In addition, RB1 is often described as a prototype for the class of tumor suppressor genes. Regulating its transcription and a negative regulator of cell proliferation called is its gene product (pRB). Cancer genome sequencing confirmed that RB1 is mutated in a variety of cancers, including small-cell lung cancers. pRB acts as a tumor suppressor, which means that it regulates cell growth and keeps cells from dividing too fast or in an uncontrolled manner. pRB stops other proteins from triggering DNA replication, the process by which DNA makes a copy of itself under specific conditions. Tight regulation of this process controls cell division and helps prevent the growth of tumors because DNA replication must occur before a cell can divide. Moreover, pRB interacts with other proteins to influence cell survival, the self-destruction of cells (apoptosis), and the process by which cells mature to carry out certain functions (differentiation).

There exists 9 LSCC hub genes in this turquoise cluster (ALS2CR3, ALPK1, STAT3, PHF11, SART3, SIDT2, MLL, RYR1, and MED11).

The alpha-protein kinase 1 gene (ALPK1), encodes this newly explored protein kinase. The fact that the expression of ALPK1 is related to inflammation and various diseases, like lung and colorectal cancer tissue has been the concentration of recent studies. Additionally, several recent studies have demonstrated this finding. ALPK1 plays a critical role in protein selection and space polarization involving myosin Ia in epithelial cells.^[34]^ It can generate an inflammatory response through the activation of nuclear factor-κb and mitogen-activated protein kinase.^[35,36]^ In a recent study designed to evaluate the correlation between ALPK1 and diabetic glomerulosclerosis, it was found that ALPK1 in atrophic renal tubules could contribute to chronic inflammation of the kidneys.^[37]^ Relatedly, an association to myocardial infarction might be attributed to the effect of vascular inflammation due to atherosclerotic plaques hindering the flow of blood into coronary arterial walls caused by the transient activation of inflammation.^[38]^ Thus, recent studies have indicated that a considerable influence on the development of inflammation in a variety of tissues could be due to the encoded ALPK1.

STAT3, a member of the STAT protein family, is considered to be involved in various processes, such as IFN-γ signaling, development of mammary glands, growth hormones response, and embryogenesis. Through controlling fundamental cellular functions including cell cycle and apoptosis, it plays a crucial role in cancer development. The deregulation of STAT3 pathway is involved in various diseases, including many types of cancer. STAT3 constitutive activation has been shown to contribute to tumor development and progression, while IL-6/JAK pathway plays a crucial role in aberrant STAT3 signaling cascades. Mounting evidence appears to indicate divergent roles in cancer biology, which could function as a potent suppressor on some occasions.^[39]^

Squamous cell carcinoma antigen recognized by T-cells 3 is a protein that in humans is encoded by the SART3 gene. The protein encoded by this gene is a RNA-binding nuclear protein that is a tumor-rejection antigen.^[40]^ Capable of inducing HLA-A24-restricted and tumor-specific cytotoxic T lymphocytes in cancer patients, this antigen possesses tumor epitopes and may be useful for specific immunotherapy. Exhibiting immunogenicity as cancer vaccines in mouse tumor models and clinical studies, these synthetic SART3 peptides bind to various mouse and human MHC haplotypes.^[41–43]^ Expression of SART3 antigen in oral cancer as a candidate of tumor antigens for use in specific immunotherapy was investigated by Fukuda. The SART3 antigen was detected in all of oral cancer cell lines tested. Therefore, the SART3 antigen might be an appropriate vaccine for oral cancer patients.^[44]^

The Ryanodine receptor 1 (RYR1) triggers the release of calcium, which causes a chain reaction that transforms ordinary breast cancer cells into cancer stem cells. RYR1 also functions as a calcium release channel in the sarcoplasmic reticulum, in addition to a connection between the sarcoplasmic reticulum and the transverse tubule. Additionally, there is an association with the dihydropyridine receptor (L-type calcium channels) within the sarcolemma of the T-tubule and RYR1. This association, in turn, opens in response to depolarization, thereby effectively meaning that the RYR1 channel opens in response to depolarization of the cell. In a related study by Semenzas, the mammary glands of mice were injected with human breast cancer cells and then the mice were treated with carboplatin after tumors had formed. The researchers reported that the loss of either glutathione S-transferase omega 1 (GSTO1) or RYR1, reduced the number of cancer stem cells in the primary tumor. This in turn blocked metastasis of cancer cells from the primary tumor to the lungs, decreased the duration of chemotherapy required to induce remission, and increased the duration of time after chemotherapy was stopped that the mice remained tumor free.^[45]^

In conclusion, an integrated bioinformatics analysis of genes that may be associated with LSCC was performed in the present study. Compared with adjacent noncancerous samples, a total of 116 DEGs were screened from LSCC samples. The evidence suggests that CCND1, LATS, and RB1may play a role in LSCC. Nevertheless, further studies are needed to reveal their specific functions in LSCC. They provide a powerful biomarker discovery platform to better understand the progression of LSCC and to uncover potential therapeutic targets in the treatment of LSCC. Improved monitoring of LSCC and resulting improvement of treatment of LSCC might stem from new information.

## 5 Author contributions

Conceived and designed the study: CY and XY.

Date download and DEG screening: SW and LW.

Bioinformatics analysis: CY, LN, and JL.

Wrote and revises the paper: CY, SW, XY, LW, LN, and JL.
